# Cross-Cultural Adaptation and Validation of the Polish Version of the Orthognathic Quality of Life Questionnaire (OQLQ)

**DOI:** 10.3390/jcm15176764

**Published:** 2026-08-31

**Authors:** Małgorzata Gałczyńska-Rusin, Maciej Okła, Martyna Wardak, Małgorzata Pobudek-Radzikowska, Małgorzata Idzior-Haufa, Agata Czajka-Jakubowska

**Affiliations:** 1 Department of Orthodontics and Temporomandibular Disorders, Poznan University of Medical Sciences, 60-812 Poznan, Poland; 2 Department of Maxillofacial Surgery, Poznan University of Medical Sciences, 61-701 Poznan, Poland; maciejokla@ump.edu.pl

**Keywords:** orthognathic quality of life questionnaire, dentofacial deformities, quality of life, cross-cultural adaptation, psychometric validation

## Abstract

**Background/Objectives**: Dentofacial deformities may adversely affect facial aesthetics, oral function, psychological well-being, and social functioning. The Orthognathic Quality of Life Questionnaire (OQLQ) is a condition-specific instrument developed to assess these effects, but no validated Polish version has previously been available. This study aimed to translate and culturally adapt the OQLQ into Polish and evaluate its reliability and construct validity, including structural validity. **Methods**: The OQLQ was translated and culturally adapted in accordance with established guidelines. The study included 161 adults: 49 patients with dentofacial deformities awaiting orthognathic surgery, 55 orthodontic patients without dentofacial deformities, and 57 untreated controls. Internal consistency was assessed using Cronbach’s alpha. Thirty participants completed the questionnaire again after one week to assess test–retest reliability using intraclass correlation coefficients (ICCs) with 95% confidence intervals (CIs). Measurement error was quantified using the standard error of measurement (SEM) and the minimal detectable change at the 95% confidence level (MDC95). Structural validity was examined by confirmatory factor analysis (CFA) of the original four-factor model using diagonally weighted least squares for ordinal items. Known-groups, convergent, and discriminant validity were evaluated using predefined hypotheses. **Results**: The Polish OQLQ demonstrated excellent internal consistency for the total score (Cronbach’s alpha = 0.957), with domain-level coefficients ranging from 0.879 to 0.948. The total-score ICC was 0.942 (95% CI, 0.88–0.97), and domain ICCs ranged from 0.766 to 0.931. For the total score, the SEM was 3.37 points and the MDC95 was 9.3 points. CFA showed mixed fit (scaled CFI = 0.960, TLI = 0.954, RMSEA = 0.125 [95% CI, 0.115–0.134]; robust SRMR = 0.080), while standardized factor loadings ranged from 0.762 to 0.997. Patients awaiting orthognathic surgery had significantly higher OQLQ scores than both comparison groups (*p* < 0.001), with large total-score effects versus the non-orthodontic control group (Hedges’ g = 1.55; 95% CI, 1.12–1.99) and the orthodontic group (g = 1.27; 95% CI, 0.85–1.69). Fourteen of the 15 predefined primary construct-validity hypotheses (93.3%) were supported. **Conclusions**: The Polish OQLQ demonstrated good-to-excellent reliability and satisfactory known-groups, convergent, and discriminant validity. The CFA provided partial rather than unequivocal support for the original four-factor structure. The instrument may be used with appropriate caution to assess condition-specific quality of life in Polish-speaking patients with dentofacial deformities; replication of its structural validity in a larger independent clinical sample and evaluation of responsiveness are warranted.

## 1. Introduction

Dentofacial deformities (DFDs) comprise skeletal discrepancies affecting the jaws, dentition, and facial proportions, resulting in functional impairment and altered facial appearance [1,2]. Patients with DFDs frequently experience difficulties with mastication, speech, and oral function, while an altered facial appearance may negatively influence self-esteem, self-confidence, and social interactions [3,4]. Consequently, DFDs have a substantial negative impact on patients’ oral health-related quality of life (OHRQoL), affecting physical, psychological, and social well-being [2,3]. Assessment of quality of life has therefore become an essential outcome measure in the management of patients with dentofacial deformities and in evaluating the effectiveness of orthognathic treatment [5,6]. Although generic health questionnaires, such as the Short-Form Health Survey (SF-36), and generic oral health questionnaires, such as the Oral Health Impact Profile (OHIP), are widely used, they are less sensitive to changes specifically related to dentofacial deformities and orthognathic treatment [1,4]. To overcome these limitations, Cunningham and colleagues developed the Orthognathic Quality of Life Questionnaire (OQLQ), a condition-specific patient-reported outcome measure designed specifically for individuals with dentofacial deformities [5]. The OQLQ consists of 22 items grouped into four domains evaluating facial esthetics, oral function, awareness of dentofacial esthetics, and the social aspects of dentofacial deformity, with higher scores indicating poorer quality of life [5]. Compared with generic instruments, the OQLQ has demonstrated superior validity, responsiveness, and sensitivity in detecting changes associated with dentofacial deformities and orthognathic treatment outcomes [7]. Because of these properties, the OQLQ has become the most widely used condition-specific questionnaire for evaluating quality of life in patients undergoing orthognathic treatment and has been translated and validated into numerous languages, including Chinese, Thai, Spanish, Portuguese, Turkish, Swedish, Dutch, and Hungarian [1,4,7,8,9,10,11,12].

Despite its widespread use worldwide, there is currently no validated Polish version of the Orthognathic Quality of Life Questionnaire (OQLQ). This absence limits standardized clinical assessment and international research involving Polish-speaking patients with dentofacial deformities. Cross-cultural adaptation cannot be assumed to preserve the original measurement structure, as differences in language, culture, clinical case mix, and the interpretation of appearance-related items have led to item redistribution or modification in previous adaptations. A validated Polish version would support routine patient-reported assessment, treatment evaluation, and comparison with international studies. Therefore, this study aimed to translate and culturally adapt the OQLQ into Polish and evaluate its reliability and construct validity. We hypothesized that the original four-factor structure would receive acceptable support; internal consistency and test–retest reliability would be at least good; patients awaiting orthognathic surgery would have higher OQLQ scores than both comparison groups; and correlations with functional and psychosocial measures would follow the predefined directions and magnitudes specified below.

## 2. Materials and Methods

### 2.1. Study Design

This study was conducted to translate, culturally adapt, and validate the Polish version of the Orthognathic Quality of Life Questionnaire (OQLQ). The study consisted of two phases: (1) cross-cultural translation and adaptation of the original English questionnaire and (2) psychometric evaluation of the Polish version.

### 2.2. Participants

Participants were recruited between May 2025 and May 2026 at the Department of Orthodontics and Temporomandibular Disorders and the Department of Maxillofacial Surgery, Poznan University of Medical Sciences, Poznan, Poland. Consecutive eligible patients who agreed to participate were enrolled in the orthodontic and orthognathic groups; the non-orthodontic group comprised consecutive eligible volunteers willing to participate. The study included three independent groups of adults: patients with dentofacial deformities scheduled for orthognathic surgery, orthodontic patients without dentofacial deformities requiring surgical correction, and non-orthodontic controls without a history of orthodontic treatment. The orthodontic group was included to distinguish the quality-of-life burden attributable to a surgically relevant dentofacial deformity from the possible influence of orthodontic treatment or orthodontic concerns alone. General inclusion criteria were age 18 years or older, native Polish language, ability to provide informed consent, and ability to complete the study questionnaires. Group-specific inclusion criteria were: (1) a skeletal dentofacial deformity requiring combined orthodontic–surgical treatment and placement on the orthognathic surgery schedule for the orthognathic group; (2) current orthodontic treatment without a dentofacial deformity requiring orthognathic surgery for the orthodontic group; and (3) no previous orthodontic treatment and no dentofacial deformity requiring surgical correction for the non-orthodontic group. Indication for orthognathic surgery was a clinically relevant skeletal jaw discrepancy that could not be adequately corrected by orthodontic treatment alone and was associated with functional and/or esthetic impairment; qualification was based on routine clinical and radiographic assessment by the treating orthodontic and maxillofacial surgery teams. Exclusion criteria were craniofacial syndromes, cleft lip and/or palate, previous orthognathic surgery, severe systemic disease affecting oral function, inability to understand the Polish questionnaires, or inability to provide informed consent. Detailed standardized classifications of deformity type and severity were not available in the study dataset and, therefore, could not be reported or used for subgroup analyses. All participants provided written informed consent. The study was approved by the Bioethics Committee of Poznan University of Medical Sciences (approval no. KB-388/25).

### 2.3. Translation and Cross-Cultural Adaptation

The translation and cross-cultural adaptation of the OQLQ were performed according to the internationally accepted guidelines proposed by Beaton et al. [13]. The adaptation process consisted of forward translation, synthesis of the translated versions, backward translation, expert committee review, pretesting, and preparation of the final Polish version.

First, the original English questionnaire was independently translated into Polish by two bilingual translators, both native Polish speakers. One translator had a medical background and was familiar with the concepts assessed by the questionnaire, whereas the other had no medical training and was blinded to the study objectives. The two forward translations were compared and synthesized into one preliminary Polish version. This version was subsequently back-translated into English by two independent native English speakers fluent in Polish who had no prior knowledge of the original questionnaire, with emphasis on conceptual rather than literal equivalence. An expert committee comprising orthodontists, specialists in temporomandibular disorders and orofacial pain, researchers experienced in patient-reported outcome measures, and professional translators used a structured expert-judgment procedure. Committee members independently evaluated semantic, idiomatic, experiential, and conceptual equivalence; all discrepancies were then discussed during a consensus meeting until unanimous agreement was reached. The pre-final Polish version was pilot tested in 12 Polish-speaking adults, including four participants from each study group, to assess clarity, comprehensibility, and cultural relevance. All participants considered the items understandable and culturally appropriate; therefore, no further linguistic modifications were required after pilot testing. The final Polish OQLQ is provided as Supplementary File S1.

### 2.4. Outcome Measures

The Orthognathic Quality of Life Questionnaire (OQLQ) is a condition-specific patient-reported outcome measure developed to assess quality of life in patients with dentofacial deformities undergoing orthodontic–surgical treatment. The questionnaire consists of 22 items grouped into four domains: facial esthetics items 1, 7, 10, 11, 14; oral function items 2–6; awareness of dentofacial esthetics/facial deformity items 8, 9, 12, 13; and social aspects of dentofacial deformity items 15–22. Each item is scored on a 5-point response scale from 0 to 4, where 0 indicates that the statement does not bother the patient or does not apply, and 4 indicates that it bothers the patient a lot. The total OQLQ score is calculated by summing all item scores and ranges from 0 to 88 points. The domain scores range from 0 to 20 for facial esthetics, 0 to 20 for oral function, 0 to 16 for awareness of dentofacial esthetics, and 0 to 32 for social aspects of dentofacial deformity. No universally accepted clinical cut-off points or severity categories have been established for the OQLQ; therefore, the questionnaire is usually interpreted as a continuous measure, with higher scores reflecting greater negative impact of dentofacial deformity on quality of life [5].

The Fonseca Anamnestic Index Polish version (FAI-PL) is a screening tool for temporomandibular disorder (TMD) symptoms, consisting of 10 items that assess pain, joint sounds, mandibular function, parafunctional activities, and psychological tension. Responses are scored as “yes,” “sometimes,” or “no,” with a total score indicating the severity of TMD symptoms [14].

Jaw function was evaluated using the Jaw Functional Limitation Scale (JFLS), which measures limitations in activities like mastication and communication on a scale of 0 to 10. Higher scores reflect greater functional limitations [15].

Depressive symptoms were assessed using the Patient Health Questionnaire-9 (PHQ-9). The PHQ-9 consists of 9 items corresponding to symptoms of depression experienced during the previous two weeks. Each item is scored from 0 to 3, giving a total score from 0 to 27, with higher scores indicating greater depressive symptom severity [16].

Anxiety symptoms were evaluated using the Generalized Anxiety Disorder-7 scale (GAD-7), which includes seven items assessing symptoms from the past two weeks. Scores range from 0 to 21, with higher scores indicating more severe anxiety [17].

Oral parafunctional behaviors were evaluated using the Oral Behaviors Checklist (OBC). This checklist assesses the frequency of behaviors such as clenching and grinding. Each of the 21 items is scored on a scale of 0 to 4, yielding a total OBC score of 0 to 84. A higher score indicates a greater frequency of oral behaviors [18,19].

Pain-related catastrophizing was measured using the Pain Catastrophizing Scale (PCS), comprising 13 items that assess thoughts and feelings about pain. Scores range from 0 to 52, with higher scores reflecting greater catastrophizing [20].

### 2.5. Psychometric Evaluation

The psychometric properties of the Polish OQLQ were evaluated in accordance with the COSMIN recommendations, including internal consistency, test–retest reliability, measurement error, structural validity, and construct validity [21]. Internal consistency was assessed using Cronbach’s alpha coefficients for the total questionnaire and each OQLQ domain. To assess test–retest reliability, the first 10 consecutively enrolled participants from each study group (orthodontic, orthognathic, and non-orthodontic; total *n* = 30) completed the Polish OQLQ again after a one-week interval. The one-week interval was selected to reduce the likelihood that participants would recall their previous responses while remaining sufficiently short to minimize the probability of genuine change in the construct being measured. Test–retest reliability was evaluated for the total score and each of the four domains using intraclass correlation coefficients (ICCs), with 95% confidence intervals (CIs) reported. Measurement error was evaluated for the same test–retest subgroup using the standard error of measurement (SEM) and the minimal detectable change at the 95% confidence level (MDC95). Structural validity was assessed by confirmatory factor analysis (CFA) of the original four-factor structure in the full sample (*n* = 161). Construct validity was evaluated by testing predefined hypotheses regarding known-groups, convergent, and discriminant validity. Known-groups validity was assessed by comparing OQLQ scores among three predefined groups: patients with dentofacial deformities awaiting orthognathic surgery, orthodontic patients without dentofacial deformities, and untreated individuals without previous orthodontic treatment. It was hypothesized that patients with dentofacial deformities would demonstrate significantly higher OQLQ scores than both comparison groups. Convergent validity was evaluated by examining correlations between OQLQ domain scores and measures of theoretically related constructs. A moderate positive correlation was expected between the oral function domain and JFLS. Weak-to-moderate positive correlations were expected between facial esthetics and PHQ-9 and GAD-7, whereas moderate positive correlations were expected between the awareness and social aspects domains and PHQ-9 and GAD-7. A weak positive correlation between oral function and FAI-PL was considered exploratory. Discriminant validity was assessed by examining correlations between OQLQ domains and measures of theoretically distinct constructs. Negligible or weak correlations (|ρ| < 0.30) were expected between all OQLQ domains and OBC. Similarly, weak correlations were expected between PCS and the facial esthetics, oral function, and awareness domains. A moderate positive correlation was expected between PCS and the social aspects domain because of their partially shared psychosocial content. Convergent and discriminant validity analyses were conducted exclusively in the orthognathic group (*n* = 49), as this group represented the target population for whom the OQLQ was originally developed. Restricting these analyses to patients with dentofacial deformities allowed the relationships between OQLQ domains and theoretically related or distinct constructs to be evaluated within the intended clinical population, while avoiding correlations driven primarily by between-group differences in the presence and severity of dentofacial deformity. Floor and ceiling effects were assessed in the orthognathic group, which represented the target clinical population, by calculating the percentages of participants obtaining the minimum and maximum possible total and domain scores. An effect was considered present when more than 15% of participants obtained the respective extreme score. A completed COSMIN Reporting Guideline checklist (version 2.0) is provided as Supplementary File S2.

### 2.6. Statistical Analysis

A total sample of at least 154 participants was targeted to ensure an adequate sample size for the psychometric evaluation of the 22-item OQLQ, corresponding to 7 participants per item and exceeding the COSMIN general threshold of 100 participants [22].

Data distributions were assessed using the Shapiro–Wilk test. Continuous variables were presented as means ± standard deviations or medians and interquartile ranges, depending on data distribution. Categorical variables were expressed as frequencies and percentages. Global differences between groups were evaluated using the Kruskal–Wallis test. When the global test was statistically significant, post hoc pairwise comparisons were performed using Mann–Whitney U tests with Bonferroni adjustment across the three pairwise comparisons. Pairwise between-group effect sizes were expressed as Hedges’ g with 95% confidence intervals. Sex distribution was compared using the chi-square test. Spearman’s rank correlation coefficients were used to evaluate construct validity, and their 95% confidence intervals were estimated using Fisher’s z transformation. Because the correlation analyses evaluated a limited set of a priori hypotheses and hypothesis support was determined primarily from the prespecified direction and magnitude rather than statistical significance alone, no additional multiplicity adjustment was applied to the correlation *p*-values; these *p*-values were treated as descriptive. No data were imputed, and analyses were performed using complete cases available for each analysis. A two-sided *p*-value < 0.05 was considered statistically significant. Test–retest reliability was assessed using intraclass correlation coefficients (ICCs). ICC values below 0.50 were considered poor, values between 0.50 and 0.75 moderate, values between 0.75 and 0.90 good, and values above 0.90 excellent. Measurement error was quantified as the standard error of measurement (SEM), calculated as SD × √(1 − ICC), where SD was the standard deviation of the OQLQ scores in the same test–retest subgroup. The minimal detectable change at the 95% confidence level (MDC95) was calculated as 1.96 × √2 × SEM. Correlation coefficients were interpreted according to their absolute magnitude as negligible (<0.10), weak (0.10–0.29), moderate (0.30–0.49), and strong (≥0.50). Hypotheses were considered supported when the direction and magnitude of the observed correlation were consistent with the predefined expectation. Statistical significance was reported but was not used as the sole criterion for hypothesis confirmation. All analyses other than CFA were performed using SPSS version 23. No separate a priori sample-size calculations were performed for the test–retest reliability or correlation analyses; their precision was evaluated using 95% confidence intervals.

For CFA, the 22 OQLQ items were treated as ordinal indicators and assigned to the four domains specified in the original questionnaire; the four latent factors were allowed to correlate. The model was estimated using diagonally weighted least squares (DWLS) with robust standard errors and a mean-adjusted, scaled and shifted chi-square test. Model fit was evaluated jointly using the scaled chi-square statistic, Comparative Fit Index (CFI), Tucker–Lewis Index (TLI), root mean square error of approximation (RMSEA) with its 95% CI, and standardized root mean square residual (SRMR). CFI and TLI values of approximately 0.95 or higher, RMSEA values of approximately 0.06 or lower, and SRMR values of approximately 0.08 or lower were used as interpretive benchmarks; conclusions were based on the pattern across indices rather than a single cut-off. CFA was conducted in Jamovi version 2.7.26.0 using the SEMLj module and the lavaan estimation engine [23,24].

## 3. Results

A total of 161 participants were included in the study, comprising 57 individuals without a history of orthodontic treatment, 55 orthodontic patients without dentofacial deformities, and 49 patients with dentofacial deformities scheduled for orthognathic surgery. The proportion of female participants was comparable across the three groups (61.4%, 56.4%, and 55.1%, respectively; *p* = 0.79). There were no significant differences in age between the groups (25.58 ± 7.96, 27.89 ± 8.51, and 27.57 ± 6.35 years, respectively; *p* = 0.127). The descriptive characteristics of the study population and questionnaire scores are presented in Table 1.

The Polish version of the OQLQ demonstrated excellent internal consistency for the total score (Cronbach’s alpha = 0.957), while domain-level coefficients ranged from 0.879 to 0.948. The test–retest subgroup comprised the first 10 consecutively enrolled participants from each of the three study groups (total *n* = 30). Test–retest reliability was good to excellent. The total-score ICC was 0.942 (95% CI, 0.88–0.97), and domain-level ICCs ranged from 0.766 to 0.931. SEM values ranged from 1.58 to 1.94 points across the four domains and were 3.37 points for the total score. The corresponding MDC95 values ranged from 4.4 to 5.4 points across the domains and were 9.3 points for the total OQLQ score (Table 2).

### 3.1. Structural Validity

In the full sample (*n* = 161), the prespecified four-factor CFA model converged without negative residual variances. The scaled model test was statistically significant (χ^2^(203) = 760, *p* < 0.001). Incremental fit indices met the prespecified benchmarks (scaled CFI = 0.960; scaled TLI = 0.954), whereas the scaled RMSEA indicated poor approximate fit (0.125; 95% CI, 0.115–0.134) and the robust SRMR was at the acceptability boundary (0.080). Standardized factor loadings were positive and high: 0.868–0.969 for facial esthetics, 0.762–0.975 for oral function, 0.826–0.971 for awareness, and 0.816–0.997 for social aspects. All freely estimated loadings were statistically significant (*p* < 0.001). Correlations between latent factors ranged from 0.534 to 0.824 (all *p* < 0.001). Taken together, the findings provided partial, but not unequivocal, support for the original four-factor structure.

### 3.2. Construct Validity

Known-groups validity was supported by significant global differences in facial esthetics (H(2) = 50.408, *p* < 0.001), oral function (H(2) = 82.003, *p* < 0.001), awareness (H(2) = 23.218, *p* < 0.001), and social aspects (H(2) = 22.556, *p* < 0.001). Bonferroni-adjusted pairwise comparisons showed that the orthognathic group scored significantly higher than the non-orthodontic control group in facial esthetics (U = 307.0, Z = −6.903), oral function (U = 49.0, Z = −8.540), awareness (U = 689.5, Z = −4.481), and social aspects (U = 663.5, Z = −4.645; all adjusted *p* < 0.003). The orthognathic group also scored significantly higher than the orthodontic group in facial esthetics (U = 585.5, Z = −4.963, adjusted *p* < 0.003), oral function (U = 315.0, Z = −6.724, adjusted *p* < 0.003), awareness (U = 774.0, Z = −3.737, adjusted *p* < 0.003), and social aspects (U = 907.0, Z = −2.869, adjusted *p* = 0.012). No significant differences were found between the non-orthodontic control and orthodontic groups in facial esthetics (U = 1554.0, Z = −0.078, adjusted *p* = 1.000), oral function (U = 1461.5, Z = −0.618, adjusted *p* = 1.000), awareness (U = 1526.5, Z = −0.240, adjusted *p* = 1.000), or social aspects (U = 1333.0, Z = −1.366, adjusted *p* = 0.516). Corresponding standardized mean differences are presented in Table 3.

No relevant floor or ceiling effects were observed in the orthognathic group (*n* = 49). Floor effects were 0.0% for facial esthetics, 2.0% for oral function, 2.0% for awareness, 6.1% for social aspects, and 4.1% for the total OQLQ score. Corresponding ceiling effects were 8.2%, 8.2%, 10.2%, 0.0%, and 0.0%, respectively. All values were below the predefined 15% threshold.

Convergent and discriminant validity analyses were conducted exclusively in the orthognathic group (*n* = 49). Fourteen of the 15 predefined primary hypotheses (93.3%) were supported. The only unsupported hypothesis concerned the association between the social aspects domain and OBC, which showed a weak-to-moderate negative correlation (ρ = −0.318, *p* = 0.026). The exploratory hypothesis concerning a weak positive association between oral function and FAI-PL was consistent with the expected direction and magnitude (ρ = 0.120), although the correlation was not statistically significant (*p* = 0.412). Results are presented in Table 4.

## 4. Discussion

This study is the first to translate, culturally adapt, and psychometrically validate the Orthognathic Quality of Life Questionnaire (OQLQ) for the Polish population. There is a clear need for such instruments because patients with dentofacial deformities have unique functional and psychosocial concerns [2,3,6]. The OQLQ was specifically developed to assess the impact of dentofacial deformities on various aspects, including facial esthetics, oral function, awareness of facial appearance, and social well-being [5]. It has been widely used as a patient-reported outcome measure in orthognathic research [1,2,3,4,5,6,7,8,9,10,11,12,25,26].

The translation and cross-cultural adaptation process adhered to internationally accepted guidelines proposed by Beaton et al. [13]. This process ensured that the original and Polish versions of the questionnaire maintained semantic, idiomatic, experiential, and conceptual equivalence. A similar methodology was employed in previous adaptations of the OQLQ into Chinese, Thai, Portuguese, Spanish, Turkish, and Swedish, facilitating direct comparisons of psychometric properties across different language versions [1,4,7,8,9,10]. The pre-final version was tested in 12 participants drawn equally from the three study groups, and no additional wording changes were required after pilot testing.

The Polish OQLQ demonstrated excellent internal consistency for the total score (Cronbach’s α = 0.957), while domain-level coefficients ranged from 0.879 to 0.948, indicating good-to-excellent internal consistency. These findings are comparable with previous cross-cultural adaptations, in which Cronbach’s α coefficients generally ranged from approximately 0.72 to 0.96. The Swedish version reported values between 0.80 and 0.90, the Chinese version between 0.78 and 0.94, and the Hungarian version between 0.72 and 0.89 across the individual domains [1,10,12]. The very high total-score coefficient may suggest some degree of item redundancy or content overlap. However, the domain-level coefficients were lower, and the items intentionally assess related manifestations of the impact of dentofacial deformity. All 22 items were therefore retained to preserve content coverage and comparability with the original instrument. Potential item redundancy should nevertheless be examined in future studies. Test–retest reliability was good to excellent, with domain-level ICCs ranging from 0.766 to 0.931 and an ICC of 0.942 for the total score. These findings indicate that the Polish OQLQ produces stable scores when administered repeatedly over a one-week interval. The results are comparable with those of the Swedish version, in which domain-level ICCs ranged from 0.81 to 0.93, and support the temporal stability of the Polish adaptation [10].

Measurement-error estimates provide additional information on the interpretation of repeated OQLQ scores. The domain-level SEM values ranged from 1.58 to 1.94 points, and the SEM for the total score was 3.37 points. The corresponding domain-level MDC95 values ranged from 4.4 to 5.4 points, while the total-score MDC95 was 9.3 points. Thus, an individual change exceeding 9.3 points in the total score is unlikely to be attributable to measurement error alone at the 95% confidence level. However, because an anchor-based minimal important change has not been established for the OQLQ, the MDC95 should not be interpreted as a threshold for clinically important improvement or deterioration.

The Thai validation proposed a shortened 16-item version of the OQLQ [4]. Four items from the awareness of dentofacial deformity domain were removed due to low content validity, and two oral function items were removed to improve domain-level internal consistency. The authors attributed these findings partly to cultural and context-specific differences in item interpretation [4]. In the Polish adaptation, no items were removed during pretesting, and all four original domains demonstrated good-to-excellent internal consistency. Retaining the complete 22-item set facilitates comparison with the original questionnaire and most other language adaptations.

The CFA yielded mixed evidence for the original dimensional structure. All items loaded strongly on their intended domains, and the scaled CFI and TLI exceeded 0.95, supporting the proposed four-factor pattern. However, the elevated scaled RMSEA and boundary-robust SRMR indicated residual model misfit. This discrepancy may partly reflect local dependence between similarly worded items and the heterogeneity of the pooled sample, which combined orthognathic patients with two comparison groups expected to have substantially lower scores. Previous exploratory analyses of other language adaptations have also reported cross-loadings or item redistribution across domains, suggesting that the exact OQLQ structure may vary across cultural and clinical contexts [1,4,7]. We therefore did not introduce post hoc correlated residuals or delete items solely to improve fit. The present CFA should be interpreted as partial support for the original structure and requires replication in a larger independent sample drawn exclusively from the intended orthognathic population.

The known-groups validity of the Polish OQLQ was supported by its ability to differentiate between predefined clinical groups. As anticipated, patients with dentofacial deformities awaiting orthognathic surgery reported significantly higher OQLQ scores than both orthodontic patients without dentofacial deformities and untreated controls, whereas no significant difference was observed between the latter two groups. These findings suggest that the Polish OQLQ is sensitive to the specific burden associated with dentofacial deformity rather than to orthodontic treatment itself. The standardized differences in total OQLQ scores were large for the orthognathic group relative to both the non-orthodontic control group (g = 1.55) and the orthodontic group (g = 1.27), indicating a substantial group-level burden. However, because no established minimal clinically important difference is available for the OQLQ, these effect sizes should not be interpreted as individual-level thresholds for clinically meaningful change. In the Swedish validation study, patients awaiting orthognathic surgery also had significantly higher total OQLQ scores than general dental patients and patients referred for third-molar removal. However, the total score did not differ significantly from that of patients who had completed orthodontic treatment, although the orthognathic group reported significantly greater limitations in the oral function domain [10]. The recent Thai validation further demonstrated differences in OQLQ scores according to deformity severity and treatment need [4]. Collectively, these findings support the OQLQ’s ability to distinguish between clinically relevant groups and indicate that the magnitude of between-group differences may depend on the characteristics and treatment status of the comparison groups.

No relevant floor or ceiling effects were identified in the orthognathic group. This finding indicates that the Polish OQLQ retained the ability to differentiate among patients across the observed range of condition-specific quality-of-life impairment, without excessive clustering at the minimum or maximum possible scores. The highest observed proportion was the ceiling effect for the awareness domain (10.2%), which remained below the predefined 15% threshold. Nevertheless, the absence of floor and ceiling effects in a cross-sectional preoperative sample does not establish responsiveness, which should be evaluated longitudinally.

Compared with most previous validation studies, the present study assessed construct validity using a broader range of theoretically relevant comparator measures. The original validation primarily evaluated convergent validity against the SF-36 and a visual analog scale, whereas subsequent language adaptations relied mainly on generic oral health questionnaires (OHIP-14), jaw function (JFLS), or facial esthetics scales [4,8,9,10,12]. In contrast, the present study simultaneously examined relationships between the OQLQ and multiple theoretically relevant constructs, including jaw functional limitation (JFLS), temporomandibular disorder symptoms (FAI-PL), depressive symptoms (PHQ-9), anxiety symptoms (GAD-7), pain catastrophizing (PCS), and oral parafunctional behaviors (OBC). This multidimensional approach allowed not only the evaluation of convergent validity but also discriminant validity based on predefined hypotheses, providing a more comprehensive psychometric assessment than most previously published language adaptations. This hypothesis-driven, multidimensional approach extended previous validation strategies by simultaneously examining functional, TMD-related, psychological, pain-related, and behavioral constructs. Nevertheless, these analyses were conducted only in the orthognathic group and should be interpreted with caution, given its limited sample size.

The moderate correlation between the oral function domain and JFLS supports its convergent validity and indicates that this domain reflects functional limitations associated with dentofacial deformity. A stronger association between the corresponding constructs was reported in the Swedish validation, where the correlation between OQLQ oral function and JFLS reached ρ = 0.71 [10]. The lower correlation observed in the present study may reflect differences in sample composition, functional severity, or the range of jaw limitations, but its direction and construct-specific pattern remain consistent with previous findings.

The strongest associations with depressive and anxiety symptoms were observed for the awareness domain, followed by the social aspects domain. This pattern supports the multidimensional nature of the OQLQ and indicates that greater concern about facial and dental appearance coexists with greater emotional distress. Similar construct-specific relationships were reported in the Hungarian validation, in which psychosocial OHIP-14 dimensions correlated particularly with the social aspects and awareness domains of the OQLQ [12]. Furthermore, Cordeiro et al. found that preoperative depression was associated with poorer OQLQ scores, supporting the clinical relevance of assessing psychological well-being alongside condition-specific quality of life [3].

Associations between OBC and the facial esthetics, oral function, and awareness domains were negligible or weak, supporting the discriminant validity of these domains. However, the social aspects domain showed an unexpected weak-to-moderate negative correlation with OBC (ρ = −0.318), contrary to the predefined hypothesis. Several alternative explanations should be considered. The OBC measures the frequency of oral behaviors rather than their perceived social or emotional consequences; therefore, the two constructs may be less closely related than initially assumed. Range restriction within the orthognathic group, differences in awareness or reporting of oral behaviors, and unmeasured psychological or clinical factors may also have influenced the observed association. Moreover, given the cross-sectional design and the number of correlations examined, a chance finding cannot be excluded. This result should therefore not be interpreted causally and requires replication in a larger clinical sample.

Correlations of PCS with the facial esthetics, oral function, and awareness domains were weak, supporting the relative distinctiveness of pain catastrophizing from these aspects of dentofacial quality of life. In contrast, PCS showed a moderate positive correlation with the social aspects domain (ρ = 0.369), consistent with the predefined hypothesis. This association may reflect partially overlapping psychosocial content. Previous research has linked pain catastrophizing with psychological distress and pain-related disability, which may help explain its stronger association with the psychosocial rather than the functional or esthetic dimensions of the OQLQ [27,28].

Several limitations should be acknowledged. First, convergent and discriminant validity analyses were restricted to the orthognathic group and were based on a relatively small sample of 49 participants; no separate a priori sample-size calculation was performed for these correlations, and the resulting confidence intervals indicate limited precision for some estimates. Second, the CFA was performed in the pooled sample rather than in an independent sample composed exclusively of patients with dentofacial deformities. Although this provided 161 observations for the 22-item model, between-group heterogeneity and low scores in the comparison groups may have influenced the polychoric correlation matrix and model-fit indices. The mixed CFA results, particularly the elevated RMSEA, preclude claiming unequivocal confirmation of the original four-factor structure. Third, a detailed standardized classification of dentofacial deformity type and severity was not available, preventing evaluation of structural or score differences across skeletal subtypes and severity levels. Fourth, recruitment was conducted within two departments of a single academic institution using consecutive eligible patients and volunteers who agreed to participate; selection related to willingness to participate, and the institutional setting may limit generalizability. Fifth, no separate a priori sample-size calculation was performed for the 30-participant test–retest analysis, and participant stability during the retest interval was not independently confirmed. Measurement invariance and responsiveness were not evaluated. Future multicenter longitudinal studies should recruit larger target-population samples, document deformity type and severity using standardized clinical and cephalometric classifications, replicate the factor structure, confirm the measurement-error estimates, evaluate measurement invariance and responsiveness, and establish an anchor-based minimal important change against which the MDC95 can be interpreted.

## 5. Conclusions

In conclusion, the Polish OQLQ demonstrated good-to-excellent internal consistency and test–retest reliability, quantifiable measurement error, and satisfactory known-groups, convergent, and discriminant validity. CFA provided partial rather than unequivocal support for the original four-factor structure: standardized loadings and incremental fit indices were strong, but RMSEA indicated residual misfit. The complete 22-item instrument may be used with appropriate caution to assess condition-specific quality of life in Polish-speaking patients with dentofacial deformities. Its structural validity should be replicated in a larger independent clinical sample, responsiveness and measurement invariance should be evaluated, and the MDC95 should be interpreted against a future anchor-based minimal important change before broader claims of full psychometric validation are made.

## Figures and Tables

**Table 1:** Characteristics of the study groups.

Variable	Non-Orthodontic	Orthodontic	Orthognathic	*p*-Value
Female, *n* (%)	35 (61.4)	31 (56.4)	27 (55.1)	0.79 *
Age	25.58 ± 7.96	27.89 ± 8.51	27.57 ± 6.35	0.127
OQLQ	17.43 ± 13.49 ^a^	20.44 ± 17.21 ^a^	45.41 ± 21.91 ^b^	<0.001
OQLQ Facial esthetics	4.90 ± 5.34 ^a^	5.48 ± 6.38 ^a^	12.52 ± 5.33 ^b^	<0.001
OQLQ Oral function	1.83 ± 2.05 ^a^	2.38 ± 2.93 ^a^	11.09 ± 5.48 ^b^	<0.001
OQLQ Awareness	5.63 ± 4.20 ^a^	5.53 ± 4.44 ^a^	9.18 ± 4.38 ^b^	<0.001
OQLQ Social aspects	5.06 ± 5.71 ^a^	7.10 ± 7.41 ^a^	13.21 ± 10.25 ^b^	<0.001
FAI	29.63 ± 19.05	30.51 ± 19.15	35.15 ± 23.35	0.291
JFLS	7.74 ± 16.77 ^a^	10.18 ± 22.87 ^a^	21.78 ± 23.04 ^b^	<0.001
OBC	26.20 ± 8.05	26.10 ± 8.36	24.57 ± 10.67	0.690
PCS	14.53 ± 11.01 ^a^	14.05 ± 11.10 ^a^	8.53 ± 5.97 ^b^	0.002
PHQ-9	8.20 ± 4.46	9.02 ± 5.32	6.60 ± 4.04	0.101
GAD-7	8.67 ± 5.21 ^a^	9.66 ± 5.98 ^a^	5.98 ± 3.83 ^b^	0.009

Values are presented as mean ± standard deviation. Global continuous-variable comparisons were performed using the Kruskal–Wallis test. Different superscript letters indicate statistically significant Bonferroni-adjusted pairwise differences; groups sharing the same letter did not differ significantly. Sex distribution was compared using the chi-square test. * Chi-square test. OQLQ, Orthognathic Quality of Life Questionnaire; FAI, Fonseca Anamnestic Index; JFLS, Jaw Functional Limitation Scale; OBC, Oral Behaviors Checklist; PCS, Pain Catastrophizing Scale; PHQ-9, Patient Health Questionnaire-9; GAD-7, Generalized Anxiety Disorder-7.

**Table 2:** Internal consistency and test–retest reliability of the Polish version of the Orthognathic Quality of Life Questionnaire (OQLQ).

Scale	Items	No. of Items	Cronbach’s α	ICC (95% CI)	SEM	MDC95
Facial esthetics	Q1, Q7, Q10, Q11, Q14	5	0.927	0.889 (0.78–0.95)	1.67	4.6
Oral function	Q2–Q6	5	0.904	0.876 (0.76–0.94)	1.58	4.4
Awareness of dentofacial deformity	Q8, Q9, Q12, Q13	4	0.879	0.766 (0.59–0.88)	1.94	5.4
Social aspects of dentofacial deformity	Q15–Q22	8	0.948	0.931 (0.86–0.97)	1.84	5.1
Total OQLQ	Q1–Q22	22	0.957	0.942 (0.88–0.97)	3.37	9.3

Cronbach’s alpha was used to assess internal consistency. The intraclass correlation coefficient (ICC) with its 95% confidence interval (CI) was used to assess test–retest reliability over a one-week interval in a subgroup comprising the first 10 consecutively enrolled participants from each study group (total *n* = 30). SEM, standard error of measurement; MDC95, minimal detectable change at the 95% confidence level.

**Table 3:** Pairwise standardized mean differences in OQLQ scores: Hedges’ g (95% CI).

OQLQ Score	Control vs. Orthodontic	Control vs. Orthognathic	Orthodontic vs. Orthognathic
Facial esthetics	0.10 (−0.27 to 0.47)	1.42 (0.99 to 1.84)	1.18 (0.77 to 1.60)
Oral function	0.22 (−0.15 to 0.59)	2.29 (1.80 to 2.78)	2.00 (1.53 to 2.47)
Awareness	−0.02 (−0.39 to 0.35)	0.82 (0.43 to 1.22)	0.82 (0.42 to 1.22)
Social aspects	0.31 (−0.06 to 0.68)	1.00 (0.59 to 1.40)	0.68 (0.29 to 1.08)
Total OQLQ	0.19 (−0.18 to 0.56)	1.55 (1.12 to 1.99)	1.27 (0.85 to 1.69)

Note: Values are Hedges’ g with 95% confidence intervals, calculated from group means, standard deviations, and sample sizes. Positive values indicate higher (poorer) OQLQ scores in the second listed group. OQLQ, Orthognathic Quality of Life Questionnaire; CI, confidence interval.

**Table 4:** Construct validity of the Polish Orthognathic Quality of Life Questionnaire (OQLQ) based on predefined hypotheses.

OQLQ Domain	Comparator	Expected Relationship	Spearman’s ρ (95% CI)	*p*-Value	Supported
Oral function	JFLS	Moderate positive	0.363 (0.091 to 0.585)	0.010	Yes
Oral function	FAI-PL	Weak positive, exploratory	0.120 (−0.167 to 0.388)	0.412	Yes *
Facial esthetics	PHQ-9	Weak-to-moderate positive	0.301 (0.022 to 0.537)	0.036	Yes
Facial esthetics	GAD-7	Weak-to-moderate positive	0.272 (−0.010 to 0.514)	0.059	Yes
Awareness	PHQ-9	Moderate positive	0.523 (0.283 to 0.701)	<0.001	Yes
Awareness	GAD-7	Moderate positive	0.504 (0.260 to 0.688)	<0.001	Yes
Social aspects	PHQ-9	Moderate positive	0.440 (0.181 to 0.642)	0.002	Yes
Social aspects	GAD-7	Moderate positive	0.394 (0.127 to 0.608)	0.005	Yes
Facial esthetics	OBC	None or weak, |ρ| < 0.30	0.042 (−0.242 to 0.319)	0.775	Yes
Oral function	OBC	None or weak, |ρ| < 0.30	0.111 (−0.176 to 0.380)	0.448	Yes
Awareness	OBC	None or weak, |ρ| < 0.30	0.052 (−0.233 to 0.328)	0.723	Yes
Social aspects	OBC	None or weak, |ρ| < 0.30	−0.318 (−0.550 to −0.040)	0.026	No
Facial esthetics	PCS	Weak, |ρ| < 0.30	0.242 (−0.042 to 0.490)	0.094	Yes
Oral function	PCS	Weak, |ρ| < 0.30	0.234 (−0.051 to 0.483)	0.106	Yes
Awareness	PCS	Weak, |ρ| < 0.30	0.246 (−0.038 to 0.493)	0.088	Yes
Social aspects	PCS	Moderate positive	0.369 (0.098 to 0.589)	0.009	Yes

Analyses were conducted exclusively in the orthognathic group (*n* = 49). Values are Spearman’s rank correlation coefficients with 95% CIs estimated using Fisher’s z transformation. Correlations were interpreted by absolute magnitude as negligible (<0.10), weak (0.10–0.29), moderate (0.30–0.49), and strong (≥0.50). Hypotheses were considered supported when the observed direction and magnitude were consistent with the predefined expectation; statistical significance was not the sole criterion. * Exploratory hypothesis. JFLS, Jaw Functional Limitation Scale; FAI-PL, Polish version of the Fonseca Anamnestic Index; PHQ-9, Patient Health Questionnaire-9; GAD-7, Generalized Anxiety Disorder-7; OBC, Oral Behaviors Checklist; PCS, Pain Catastrophizing Scale.

## Data Availability

The data presented in this study are available from the corresponding author upon reasonable request. The data are not publicly available due to participant privacy and ethical restrictions.

## Supplementary Materials

The following supporting information can be downloaded at https://www.mdpi.com/article/10.3390/jcm15176764/s1, File S1: Polish version of the OQLQ; File S2: Completed COSMIN Reporting Guideline checklist (version 2.0) [29].

## Abbreviations

The following abbreviations are used in this manuscript:

CFA	Confirmatory Factor Analysis
CFI	Comparative Fit Index
COSMIN	Consensus-Based Standards for the Selection of Health Measurement Instruments
DFD	Dentofacial Deformity
DWLS	Diagonally Weighted Least Squares
FAI-PL	Polish Version of the Fonseca Anamnestic Index
GAD-7	Generalized Anxiety Disorder-7
ICC	Intraclass Correlation Coefficient
JFLS	Jaw Functional Limitation Scale
OBC	Oral Behaviors Checklist
OHIP-14	14-Item Oral Health Impact Profile
OHRQoL	Oral Health-Related Quality of Life
OQLQ	Orthognathic Quality of Life Questionnaire
PCS	Pain Catastrophizing Scale
PHQ-9	Patient Health Questionnaire-9
RMSEA	Root Mean Square Error of Approximation
SF-36	36-Item Short-Form Health Survey
SRMR	Standardized Root Mean Square Residual
TLI	Tucker–Lewis Index
TMD	Temporomandibular Disorder
