# Supplementary material for: Cross-Cultural Adaptation and Validation of the Polish Version of the Orthognathic Quality of Life Questionnaire (OQLQ)
Source: J Clin Med. 2026 Aug 31;15(17):6764. doi: 10.3390/jcm15176764 (PMC13565885; doi:10.3390/jcm15176764)
Supplement: Supplementary file 1 [file jcm-15-06764-s001.zip › jcm-4480752-File S1.pdf]

Kwestionariusz OQLQ – Wersja polska

Prosimy o uważne przeczytanie poniższych stwierdzeń i zaznaczenie, w jakim stopniu każde z nich Ci przeszkadza. 1 oznacza, że dana kwestia przeszkadza Ci w niewielkim stopniu, a 4 – że przeszkadza Ci w bardzo dużym stopniu. Wartości 2 i 3 oznaczają stopnie pośrednie. N/D oznacza, że dane stwierdzenie Cię nie dotyczy lub dana kwestia wcale Ci nie przeszkadza.

Nr	Pytanie	1	2	3	4	N/D
1	Czuję się skrępowany(a) wyglądem moich zębów.	☐	☐	☐	☐	☐
2	Mam problemy z gryzieniem	☐	☐	☐	☐	☐
3	Mam problemy z żuciem.	☐	☐	☐	☐	☐
4	Są pokarmy, których unikam, ponieważ sposób, w jaki moje zęby się stykają, utrudnia ich jedzenie.	☐	☐	☐	☐	☐
5	Nie lubię jeść w miejscach publicznych.	☐	☐	☐	☐	☐
6	Miewam bóle twarzy lub szczęk.	☐	☐	☐	☐	☐
7	Nie lubię oglądać swojej twarzy z profile (widoku z boku).	☐	☐	☐	☐	☐
8	Spędzam dużo czasu przyglądając się swojej twarzy w lustrze.	☐	☐	☐	☐	☐
9	Spędzam dużo czasu przyglądając się swoim zębom w lustrze.	☐	☐	☐	☐	☐
10	Nie lubię, gdy ktoś robi mi zdjęcia.	☐	☐	☐	☐	☐
11	Nie lubię, gdy jestem nagrywany(a) na wideo.	☐	☐	☐	☐	☐
12	Często przyglądam się zębom innych ludzi.	☐	☐	☐	☐	☐
13	Często przyglądam się twarzom innych ludzi.	☐	☐	☐	☐	☐
14	Czuję się skrępowany(a) wyglądem swojej twarzy.	☐	☐	☐	☐	☐
15	Staram się zakrywać usta, gdy spotykam kogoś po raz pierwszy.	☐	☐	☐	☐	☐
16	Martwię się, kiedy mam spotkać kogoś po raz pierwszy	☐	☐	☐	☐	☐
17	Obawiam się, że ludzie będą wypowiadać przykre uwagi na temat mojego wyglądu.	☐	☐	☐	☐	☐
18	Brakuje mi pewności siebie w sytuacjach towarzyskich.	☐	☐	☐	☐	☐
19	Nie lubię się uśmiechać, gdy spotykam innych ludzi.	☐	☐	☐	☐	☐
20	Czasami czuję się przygnębiony(-a) z powodu swojego wyglądu.	☐	☐	☐	☐	☐
21	Czasami myślę, że ludzie się na mnie gapią.	☐	☐	☐	☐	☐
22	Uwagi dotyczące mojego wyglądu naprawdę mnie denerwują, nawet gdy wiem, że ludzie tylko żartują.	☐	☐	☐	☐	☐
