# Supplementary material for: Cross-Cultural Adaptation and Validation of the Polish Version of the Orthognathic Quality of Life Questionnaire (OQLQ)
Source: J Clin Med. 2026 Aug 31;15(17):6764. doi: 10.3390/jcm15176764 (PMC13565885; doi:10.3390/jcm15176764)
Supplement: Supplementary file 1 [file jcm-15-06764-s001.zip › jcm-4480752-File S2.pdf]

Completed COSMIN Reporting Guideline Checklist (Version 2.0)

Cross-Cultural Adaptation and Validation of the Polish Version of the Orthognathic Quality of Life Questionnaire (OQLQ)

Completion note. The COSMIN Reporting Guideline 2.0 is modular. All general items and all measurement-property modules are shown below. Properties not evaluated in the study are marked 'N/A - not assessed'. Status entries describe reporting in the current manuscript and do not constitute a COSMIN risk-of-bias or methodological-quality score. Floor and ceiling effects, which are not a separate item in the 68-item guideline, are reported for the orthognathic target group on p. 8 and interpreted on p. 11. Guideline source: Gagnier et al., Quality of Life Research (2025), doi:10.1007/s11136-025-03950-x.

Pagination note. *Page references correspond to the current 16-page revised manuscript file reviewed on 16 August 2026 and should be updated if final pagination changes.*

General reporting recommendations

Item	Topic	Status	Location in manuscript	Manuscript mapping / note
T1	Title	Reported	p. 1, Title	The title identifies a Polish cross-cultural adaptation and validation study of the OQLQ, a quality-of-life PROM.
A1	Objectives	Reported	pp. 1-2, Abstract	The PROM, target construct, study population, and evaluated properties are stated.
A2	Design	Reported	pp. 1-2, Abstract	The translation/adaptation phase and psychometric validation sample are summarized.
A3	Methods	Reported	pp. 1-2, Abstract	Methods for internal consistency, test-retest reliability, measurement error, CFA, and construct validity are provided.
A4	Results	Reported	pp. 1-2, Abstract	Main results are provided for reliability, measurement error, CFA, known-groups validity, and hypothesis testing.
A5	Implications / conclusions	Reported	p. 2, Abstract	Use of the Polish OQLQ is recommended with caution, and further structural-validity and responsiveness work is proposed.
I1	PROM	Reported	p. 2, Introduction	The OQLQ and the condition-specific quality-of-life construct are described.
I2	Target population and context	Reported	p. 2, Introduction	The intended population comprises individuals with dentofacial deformities undergoing orthognathic treatment.
I3	State of knowledge and rationale	Reported	p. 2, Introduction	Existing international adaptations, the lack of a validated Polish version, and the need for the study are explained.
I4	Objectives	Reported	p. 2, Introduction	The translation, cultural adaptation, and psychometric objectives and hypotheses are stated.
GM1	Study design	Reported	p. 3, Study design	Two phases are specified: cross-cultural translation/adaptation and psychometric evaluation.
GM2	Participants	Reported	pp. 3-4, Participants	Recruitment period, centers, consecutive recruitment, group definitions, and inclusion/exclusion criteria are described.
GM3	PROM details	Partly reported	p. 4, Outcome measures	The language, 22-item/four-domain structure, response scale, direction, ranges, and scoring are described. The reflective/formative framework and detailed administration mode/instructions are not explicitly stated.
GM4	Additional data collection	Partly reported	pp. 4-5, Outcome measures; Psychometric evaluation	Comparator instruments and the rationale for the comparison groups and validity analyses are described; administration mode is not specified.
GM5	Time points and procedures	Partly reported	p. 5, Psychometric evaluation	A one-week OQLQ retest is reported. The timing of all comparator questionnaires is not explicitly detailed.
GM6	Sample-size justification	Partly reported	pp. 5-6, Statistical analysis	The overall target (at least 154; seven participants per item) is justified. No separate a priori calculation was performed for the test-retest or correlation analyses.
GM7	Statistical analyses	Reported	pp. 5-6, Statistical analysis	Analyses, interpretive criteria, multiplicity approach, software, CFA estimator/fit criteria, and formulas for SEM and MDC95 are described.
GM8	Missing-data approach	Reported	p. 6, Statistical analysis	No imputation was performed; complete cases available for each analysis were used.

Item	Topic	Status	Location in manuscript	Manuscript mapping / note
**GM9**	Unplanned analyses	Not reported	Not reported	The manuscript does not identify whether any analyses were unplanned or added after the original analysis plan.
**GR1**	Participant characteristics	Partly reported	pp. 6-7, Table 1	Age, sex, group sizes, and questionnaire scores are reported by group. Standardized deformity type/severity was unavailable.
**GR2**	Sample size	Reported	pp. 6-9, Results	The total sample and analysis-specific samples (CFA n=161; test-retest n=30; construct validity n=49) are reported.
**GR3**	Missing data	Not reported	Not reported	The amount and reasons for missing data are not presented in the Results.
**GR4**	Results for objectives	Reported	pp. 6-9, Results; Tables 1-4	Results are provided for internal consistency, reliability, measurement error, structural validity, known-groups validity, and convergent/discriminant validity.
**DC1**	Evidence for measurement properties	Reported	pp. 9-13, Discussion	Main findings are interpreted for each assessed property, including SEM/MDC95; CFA support is explicitly described as partial.
**DC2**	Practical relevance	Reported	pp. 10-13, Discussion; Conclusions	Clinical/research use is discussed with caution; MDC95 and group effect sizes are not equated with individual clinical importance.
**DC3**	Strengths and limitations	Reported	pp. 12-13, Discussion	Limitations related to sample size, pooled CFA, deformity classification, recruitment, participant stability, and unassessed properties are discussed.
**DC4**	Generalizability	Reported	pp. 12-13, Limitations	Single-institution recruitment, willingness to participate, and pooled-sample heterogeneity are considered.
**DC5**	Instrument changes	Reported	pp. 4 and 10, Translation; Discussion	No linguistic changes or item deletion were required after pilot testing; all 22 items were retained.
**DC6**	Future research	Reported	p. 13, Limitations	Independent structural replication, confirmation of measurement-error estimates, invariance, responsiveness, and anchor-based minimal important change are proposed.
**DC7**	Conclusions	Reported	p. 13, Conclusions	Conclusions are restricted to the evaluated properties and recommend cautious use.
**O1**	Conflict of interest	Reported	p. 13, Conflicts of Interest	The authors declare no conflicts of interest.

Content validity

Item	Topic	Status	Location in manuscript	Manuscript mapping / note
**CV1**	Relevance - methods	Partly reported	pp. 3-4, Translation and cross-cultural adaptation	Experts assessed semantic, idiomatic, experiential, and conceptual equivalence, and participants assessed cultural relevance. Item-by-item procedures for instructions, response options, and any recall period are not detailed.
**CV2**	Comprehensiveness - methods	Not reported	Not reported	The pilot is not described as explicitly asking whether all key concepts were covered by the OQLQ.
**CV3**	Comprehensibility - methods	Partly reported	p. 4, Translation and cross-cultural adaptation	The 12-person pilot assessed clarity and comprehensibility; separate evaluation of instructions, response options, and recall period is not specified.
**CV4**	Relevance - results	Partly reported	p. 4; p. 10, Discussion	Participants considered the items culturally appropriate and no wording changes were required; separate results for all PROM components are not shown.
**CV5**	Comprehensiveness - results	Not reported	Not reported	No explicit result is provided on whether patients or professionals considered all key concepts to be represented.
**CV6**	Comprehensibility - results	Reported	p. 4; p. 10, Discussion	All pilot participants considered the items understandable; no further linguistic modifications were required.

Structural validity

Item	Topic	Status	Location in manuscript	Manuscript mapping / note

Item	Topic	Status	Location in manuscript	Manuscript mapping / note
**SV1**	Rationale for approach	Reported	p. 2; p. 5, Psychometric evaluation	CFA was selected to test the prespecified original four-factor structure.
**SV2**	Statistical analyses	Partly reported	pp. 5-6, Statistical analysis	The four-factor item allocation, correlated factors, ordinal treatment, DWLS estimation, robust test, fit indices, benchmarks, and software are reported. The correlation-matrix type and exact software/module version are not clearly specified in Methods.
**SV3**	Results	Partly reported	p. 7, Structural validity	Model-fit indices and domain-specific loading ranges are provided. All 22 item-level standardized loadings are not tabulated.

Internal consistency

Item	Topic	Status	Location in manuscript	Manuscript mapping / note
**IC1**	Methods	Partly reported	pp. 5-6, Psychometric evaluation; Statistical analysis	Cronbach's alpha and CFA-based dimensional evidence are described. Local item dependence was not formally evaluated.
**IC2**	Results	Reported	p. 7, Table 2	Cronbach's alpha is reported for each domain and the total OQLQ score.

Cross-cultural validity / measurement invariance

Item	Topic	Status	Location in manuscript	Manuscript mapping / note
**CCV1**	Group variable	N/A - not assessed	p. 13, Limitations	Measurement invariance was not evaluated. Translation/cultural adaptation alone is not a measurement-invariance analysis.
**CCV2**	Rationale for approach	N/A - not assessed	p. 13, Limitations	No multi-group invariance or differential-item-functioning analysis was conducted.
**CCV3**	Statistical analyses	N/A - not assessed	p. 13, Limitations	No measurement-invariance analysis was conducted.
**CCV4**	Results	N/A - not assessed	p. 13, Limitations	No measurement-invariance results are reported.

Reliability

Item	Topic	Status	Location in manuscript	Manuscript mapping / note
**R1**	Patient stability - methods	Partly reported	p. 5, Psychometric evaluation	A short one-week interval is specified, but clinical stability was not verified with a transition question or reassessment.
**R2**	Statistical analyses	Partly reported	pp. 5-6, Statistical analysis	ICC analysis and interpretive thresholds are described, but the specific ICC model/formula and its justification are not reported.
**R3**	Sources of variation	Partly reported	p. 5, Psychometric evaluation	Repeated self-administration over one week is described; intended sources of variation are not explicitly formulated.
**R4**	Time interval	Partly reported	p. 5, Psychometric evaluation	The one-week interval is stated, but its appropriateness is not explicitly justified.
**R5**	Patient stability - results	Not reported	Not reported	No empirical confirmation of participant stability between administrations is presented.
**R6**	Reliability results	Partly reported	p. 7, Table 2	ICCs with 95% confidence intervals are provided for all domains and the total score; variance components are not reported.

Measurement error

Item	Topic	Status	Location in manuscript	Manuscript mapping / note
**ME1**	Patient stability - methods	Partly reported	p. 5, Psychometric evaluation	Measurement error used the same 30-person test-retest subgroup and one-week interval; clinical stability was not independently verified.
**ME2**	Statistical analyses	Reported	p. 6, Statistical analysis	SEM was calculated as $SD \times \sqrt{(1 - ICC)}$, using the subgroup SD, and MDC95 as $1.96 \times \sqrt{2} \times SEM$.

Item	Topic	Status	Location in manuscript	Manuscript mapping / note
**ME3**	Sources of variation	Partly reported	p. 5, Psychometric evaluation	Repeated self-administration of the OQLQ in the same subgroup is described; additional sources of variation were not decomposed.
**ME4**	Time interval	Partly reported	p. 5, Psychometric evaluation	The one-week interval is reported, but its appropriateness is not explicitly justified.
**ME5**	Patient stability - results	Not reported	p. 13, Limitations	No empirical confirmation of participant stability between administrations was available; this is acknowledged as a limitation.
**ME6**	Measurement-error results	Reported	p. 7, Results; Table 2	SEM and MDC95 are reported for all four domains and the total OQLQ score.

Criterion validity

Item	Topic	Status	Location in manuscript	Manuscript mapping / note
**CriV1**	Criterion	N/A - not assessed	Not applicable	Criterion validity was not assessed because no accepted gold-standard measure for the OQLQ construct was used.
**CriV2**	Statistical analyses	N/A - not assessed	Not applicable	No criterion-validity analysis was conducted.
**CriV3**	Results	N/A - not assessed	Not applicable	No criterion-validity results are reported.

Hypotheses testing for construct validity

Item	Topic	Status	Location in manuscript	Manuscript mapping / note
**ConV1**	Hypotheses	Reported	p. 5, Psychometric evaluation	Directional and magnitude-based hypotheses are predefined for known-groups, convergent, and discriminant validity, with construct-based rationales.
**ConV2**	Statistical analyses	Reported	pp. 5-6, Statistical analysis	Kruskal-Wallis, Bonferroni-adjusted Mann-Whitney tests, Hedges' g, Spearman correlations, confidence intervals, and hypothesis-support criteria are specified.
**ConV3**	Results	Reported	pp. 8-9, Construct validity; Tables 3-4	All prespecified comparisons and correlations are reported, and support for each primary hypothesis is indicated.

Responsiveness

Item	Topic	Status	Location in manuscript	Manuscript mapping / note
**Resp1**	Hypotheses	N/A - not assessed	p. 13, Limitations; Conclusions	Responsiveness was not evaluated in this cross-sectional preoperative validation.
**Resp2**	Intervention / exposure	N/A - not assessed	p. 13, Limitations; Conclusions	No longitudinal intervention/exposure analysis was conducted.
**Resp3**	Statistical analyses	N/A - not assessed	p. 13, Limitations; Conclusions	No responsiveness analysis was conducted.
**Resp4**	Results	N/A - not assessed	p. 13, Limitations; Conclusions	No responsiveness results are reported; future longitudinal evaluation is recommended.
